# 

**Published:** 1889-01-05

**Authors:** 


**Notice to Advertisers and Subscribers.**

All Advertisements, Orders for Copies of The Hospital, and Business Communications should be addressed to The Publisher, *not to the Editor*, The Hospital, 38, Old Jewry, E.C.

**To Residents, Secretaries, and Matrons.**

The Editor will be glad to have the Regulations and each Report as issued by Asylums, Hospitals, Convalescent and Nursing Institutions, as well as those of other Charities, and an early copy in duplicate of all Appeals and Festival Notices, etc., addressed to *The Editor, The Lodge, Porchester Square, London, W.* Any superintendent, secretary, matron, or other chief official who regularly sends such information may be registered, on application to the Publisher, to receive free of cost a cover for binding The Hospital in half-yearly volumes.

A Scale of Charges will be found on the first page of the Nursing Supplement.

### The Student's Column.

### UNIVERSITY OF LONDON.—B.S. EXAMINATION, 1888

The following are the successful candidates in order of merit:

SURGERY.

FIRST CLASS.

Dean, Henry Percy, B.Sc. (Scholarship and Gold Medal), Univer. Coll.
Parkin, Alfred (Gold Medal), Guy's Hospital.
Starling, Ernest Henry, Guy's Hospital.
Kanthack, Alfredo A., B.A.B.Sc., Univ. Coll. L'pool & St. Barth's.
Ashworth, Percy, B.Sc., Owens Coll. & Manchester R. Infirm.

SECOND CLASS.

{ Crook, Herbert Evelyn, Guy's Hospital.
{ Thompson, James Edwin, Owens College.
Goodall, Edward Wilberforce, M.D., Guy's Hospital.

N.B.—The bracket denotes equality of merit.

### SCRAPS AND GLEANINGS.

THERE are 90 cases of smallpox at Messina just now.

THE death rate at Panama just now is about 98 per 1,000.

SIR E. GUINNESS has given £100 to the Dublin Adelaide Hospital.

WATERFORD DISPENSARY has removed to its new premises in Peter Street.

TORLINGTON appeals for funds to build a much-needed district hospital.

IT is proposed to establish a dental school in connexion with Guy's Hospital.

THE late bazaar at Twickenham resulted in £170 for St. John's Hospital.

THE extension of Brentwood County Asylum to hold 462 males is now complete.

THE Earl of Chesterfield has sent a present of pheasants to Hereford Infirmary.

THE death is announced of Dr. Carl Zeiss, the famous maker of microscopes.

THE annual meeting of the Pathological Society will be held on January 15th.

A CHILDREN'S fancy ball, in aid of the Kingstown Hospital, will be given on January 10th.

THE series of clinical manuals published by Messrs. Cassells are to be translated into Spanish.

DUNDEE ROYAL HOSPITAL has lately received a legacy of £1,000 from the late Mr. John Sharp.

IT is proposed to endow a bed in Wigan Infirmary in memory of the late Dr. Perrin, of Leigh.

MR. FREDERICK TREVES and Mr. Oppenshaw have gone on a cruise to the North Sea Fisheries.

DR. JOSEPH WIGLESWORTH has been appointed medical superintendent of Rainhill County Asylum.

GORLESTON COTTAGE HOSPITAL has had nine in-patients during the nine months it has been open.

THE Chairman of Anniesland Fever Hospital appeals for toys and books for the children's ward.

BRISTOL Royal Infirmary expenditure for 1888 will reach £12,000. The subscriptions have fallen off £150.

ST. BARNABAS COTTAGE HOSPITAL, Saltash, is under the management of the sisters of St. Mary's, Wantage.

AN amateur dramatic performance, in aid of the City of Dublin Hospital, was given on December 19th.

THE Dublin Hospital for Incurables has not a single empty bed, and nine cancer cases waiting for admission.

THE Emperor of Brazil has conferred on Dr. R. H. Gunning the Grand Dignitary of the Order of the Rose.

MR. J. ELLIOT SQUARE has been appointed surgeon at Plymouth Royal Infirmary in place of Mr. Eccles, resigned.

*Truth* has been finding fault with Mr. Walter Austin for not issuing accounts of his London Cottage Mission funds.

SIR THOMAS CLERK presided at the 103rd annual meeting of the Society for the Relief of the Destitute Sick of Edinburgh.

DR. MORISON has written to the *Lancet* defending the present provident system in vogue at the Metropolitan Hospital.

ON January 18th the medical students' dinner of Yorkshire College will be held at Leeds, Dr. C. J. Collingworth in the chair.

MRS. DYSON PERRINS has opened a convalescent home at Malvern, and offered to take suitable cases from Worcester Infirmary.

THE measles cases at Liverpool have to be sent to Brownlow Hill, as there is no accommodation for them at the town hospitals.

MR. AND MRS. HORATIO BOTTOMLY visited the Great Northern Central Hospital last week, and left a cheque for £100 behind them.

THE *Scotsman* of December 19th speaks rather doubtfully about Dr. Barnardo's missions, and his desire to enlist Scotch sympathy.

THE Marquis of Breadalbane has been awarded the medal of the Royal Humane Society for rescuing a man from drowning in the Tay.

THE *British Medical Journal* for December 15th contained an engraving and account of Anderson's College Medical School, Glasgow.

AN inquest was held at St. Bartholomew's Hospital on December 20th, on the body of a boy, aged 12, who died from swallowing the nib of a pen.

THE Baroness Burdett-Coutts gave an excellent address at the opening of the sale of work last week, in aid of the Great Northern Central Hospital.

THE prize for an ambulance offered by the Empress Augusta, will be awarded next June at Berlin, when there will be an exhibition of surgical appliances.

THE Hull Hospital Sunday Fund has been divided as follows: Infirmary, £450; Dispensary, £59; Children's Hospital, £49; Homœopathic, £16.

THE closing of Thoresby Street, Leeds, in consequence of the Infirmary extension is likely to lead to the making of a new and much more convenient road.

DR. SUTHERLAND has printed his paper read before the British Medical Association last August, under the title of "The Breakdown of the Hospital System."

EXPERIMENTS were made by volunteers at Hampstead Heath last week with the object of testing the capacity of small electric hand-lamps in searching for wounded men left after a battle. The tests proved fairly successful.

THE Christmas double number of the *Charity Record* contains a special article entitled, "Royalty and Charity," giving the most complete list yet published of the medical and other charities with which the Queen and the other members of the Royal family are more or less actively identified.

### AMUSEMENTS AND RELAXATION.

### NEW PUZZLE PRIZE SCHEME.

SECOND QUARTERLY COMPETITION COMMENCES JANUARY 5TH, ENDS MARCH 30TH, 1889.

1. Prizes will be awarded for the best Original Puzzles in Prose or Verse relating to any of the Social, Political, or Philanthropical topics of the day. Competitors are requested to *strictly* observe this rule, or their contributions will be discredited.
2. Puzzles may take any form which the ingenuity of the competitors suggest. Answers must accompany contributions, and the source of obscure or ambiguous terms be given.
3. No competitor will be allowed to take more than one prize during the Quarter.
4. Eight Prizes of Standard Books will be given, the choice of the book to be left to the winner. First, Second, Third, Fourth, and Fifth Prizes for best Original Puzzles, £1 1s., 15s., 10s. 6d., 7s. 6d., and 5s. each.
5. First, Second, and Third Prize for Solutions of 15s., 10s. 6d., and 5s. each.

N.B.—All contributions must reach the office, 38, Old Jewry, E.C., not later than the *First Post* on *Thursday* in each week.

### PUZZLES FOR SOLUTION.

**First Week.**

No. 1. DOUBLE ACROSTIC.

Ice and snow, frost and thaw,
'Jolly weather' this is reckon'd!
If my first you like not raw,
Rub and pickle with my second.

A queer old thing. You'll buy it, shall you ?

'Tis old and queer; yes, that's its value.

'The dignity of service' does the name

Much matter, if the object is the same ?

Lo! now, its cerement laid aside,
It rises quicken'd glorified.

That ghost will trouble you no more;
The hen! she cackleth at the door.

It rises; falls; now up, now down;
The tilt is a Bulgarian crown.

This it may be, yet not just;
Not what I ought, but what I must.

Like a star, yet not a star,

"How I wonder what you are!"

Embodiment of malice, envy, guile,
Instilling poison of suspicions vile.

This science takes within its range,
The little symbols of exchange.

PASTEUR.

No. 2. CONUNDRUM.—Why is the Government like a man bringing an action against his brother ? GRETTA.

No. 3. DIAMOND PUZZLE.

. 1. A consonant by some ignored.
. . . 2. A well-known health resort abroad.
. . . . . 3. A bunch of these how lovely to the sense.
. . . . . . . 4. More falls for those who just commence.
. . . . . . . . . 5. Lost, despaired of, found again.
. . . . . . . 6. In Arthur's court an officer of fame.
. . . . . 7. A lady fair who in her island home,
And round it fearless, aye was want to roam.
. . . 8. Small though I be, I wield enormous power.
. 9. A consonant, a question to which some cower

**Marks.**

| Names. | No. of Puzzles sent in Dec. 27. | No. of Marks Dec. 27. | Total of Marks. | Names. | No. of Puzzles sent in Dec. 27. | No. of Marks Dec. 27. | Total of Marks. |
|---|---|---|---|---|---|---|---|
| Patience...... | 3 | 5 | 63 | Hercules...... | — | — | 11 |
| Lady Clara... | 3 | 7 | 78 | Gretta......... | 3 | 5 | 58 |
| Tinie ......... | 3 | 6 | 71 | Dandy Dick | — | — | 5 |
| Bijou ......... | 2 | 5 | 42 | Boadicea ... | — | — | 7 |
| Thanet ...... | — | — | 30 | Nipper......... | 3 | 5 | 54 |
| G. D. ......... | — | — | 15 | Morico......... | 3 | 6 | 61 |
| Daisy ......... | — | — | 23 | A. M. E....... | — | — | 3 |
| Salford ...... | — | — | 6 | John Gilpin | — | — | 4 |

**Answers to Tenth Week Puzzles.**

No. 25. MUSICAL PUZZLE.

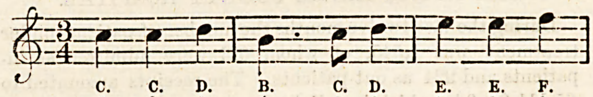

C. 1. C. 2. D. 3. B. 4. C. 5. D. 6. E. 7. E. 8. F. 9.

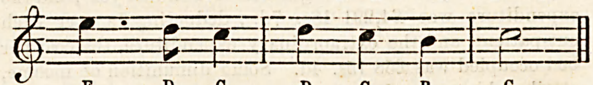

E. 10. D. 11. C. 12. D. 13 C. 14. B. 15. C. 16.

1, Cybele; 2, Charles I.; 3, Dante; 4, Bacchus; 5, Cocytus; 6, Decemviri; 7, Elysium; 8, England; 9, Fortune; 10, Erebus; 11, Discordia; 12, Cupid; 13, Delos; 14, Cyclops; 15, Bellona; 16, Comet.

(Or, First Part of God Save the Queen.)

No. 26. CHARADE.—Ho-Li-Day = Holiday.

No. 23. DOUBLE ACROSTIC.—
F orcep S
R oo K
O lympi A
S ue T
T id E

**Marks for Solution of Puzzles.**

Total Marks.—Ruffles (1), 15; Daisy, 7; Lady Clara (2), 16; Reldas (2), 13; Mater (2), 9; Condorcet, 7; Tinie 12; Gentian (2), 18; Marguerite, 5; Patience, 7; Gretta, 4; Amicus, 7; Morico, 3; Scrooge (2). 17; Smut, 2; Thanet (1), 18; Jenny Wren 3; Lauriston, 1.

The names of the winners of the First Quarterly Prizes will be given next week.

*The Figures in parentheses are Marks for the week ending Dec. 27.*

[For Conditions, see THE HOSPITAL for December 15th, 1888.]

